# Antiviral Efficacy and Host Immune Response Induction during Sequential Treatment with SB 9200 Followed by Entecavir in Woodchucks

**DOI:** 10.1371/journal.pone.0169631

**Published:** 2017-01-05

**Authors:** Manasa Suresh, Kyle E. Korolowicz, Maria Balarezo, Radhakrishnan P. Iyer, Seetharamaiyer Padmanabhan, Dillon Cleary, Rayomand Gimi, Anjaneyulu Sheri, Changsuek Yon, Bhaskar V. Kallakury, Robin D. Tucker, Nezam Afdhal, Stephan Menne

**Affiliations:** 1 Department of Microbiology & Immunology, Georgetown University Medical Center, Washington, District of Columbia, United States of America; 2 Spring Bank Pharmaceuticals, Inc., Milford, Massachusetts, United States of America; 3 Department of Pathology, Georgetown University Medical Center, Washington, District of Columbia, United States of America; 4 Department of Comparative Medicine, Georgetown University Medical Center, Washington, District of Columbia, United States of America; Indiana University, UNITED STATES

## Abstract

SB 9200, an orally bioavailable dinucleotide, activates the viral sensor proteins, retinoic acid-inducible gene 1 (RIG-I) and nucleotide-binding oligomerization domain-containing protein 2 (NOD2) causing the induction of the interferon (IFN) signaling cascade for antiviral defense. The present study evaluated the overall antiviral response in woodchucks upon induction of immune response, first with SB 9200 followed by Entecavir (ETV) *versus* reduction of viral burden with ETV followed by SB 9200 immunomodulation. Woodchucks chronically infected with woodchuck hepatitis virus (WHV) were treated orally with SB 9200 (30 mg/kg/day) and ETV (0.5 mg/kg/day). Group 1 received ETV for 4 weeks followed by SB 9200 for 12 weeks. Group 2 received SB 9200 for 12 weeks followed by ETV for 4 weeks. At the end of treatment in Group 2, average reductions of 6.4 log_10_ in serum WHV DNA and 3.3 log_10_ in WHV surface antigen were observed whereas in Group 1, average reductions of 4.2 log_10_ and 1.1 log_10_ in viremia and antigenemia were noted. Both groups demonstrated marked reductions in hepatic WHV nucleic acid levels which were more pronounced in Group 2. Following treatment cessation and the 8-week follow-up, recrudescence of viral replication was observed in Group 1 while viral relapse in Group 2 was significantly delayed. The antiviral effects observed in both groups were associated with temporally different induction of IFN-α, IFN-β, and IFN-stimulated genes in blood and liver. These results suggest that the induction of host immune responses by pretreatment with SB 9200 followed by ETV resulted in antiviral efficacy that was superior to that obtained using the strategy of viral reduction with ETV followed by immunomodulation.

## Introduction

Chronic infection with hepatitis B virus (HBV) is a major health problem and responsible for approximately 1.2 million deaths per year worldwide [1]. It is estimated that more than 2 billion individuals have serological evidence of previous or current HBV infection, and that at least 248 million are chronic carriers of HBV [1–3]. HBV carriers are at a higher risk of developing chronic hepatitis, hepatic cirrhosis, and hepatocellular carcinoma (HCC). Although safe and effective prophylactic vaccines exist, improvements in antiviral and/or immunotherapeutic strategies for the treatment of established chronic HBV infection are urgently needed. Current antiviral therapies for chronic hepatitis B (CHB) are limited to nucleos(t)ides and interferon-alpha (IFN-α) which require prolonged administration for reducing viral load and for improving the long-term outcome of CHB, but rarely lead to a cure [4]. Use of these antivirals is further limited due to the emergence of drug-resistant variants during treatment, the risk of relapse upon treatment discontinuation, and unwarranted side effects [4].

CHB is associated with a deficient and/or an inadequate host immune response against HBV since viral proteins interfere with the functions of cellular viral sensors such as retinoic acid-inducible gene 1 (RIG-I) and melanoma differentiation-associated protein 5 (MDA5) thereby disabling the innate and adaptive immune responses. For example, the HBV polymerase inhibits the activation of RIG-I in hepatocytes through interference with phosphorylation and nuclear translocation of IFN regulatory factor 3 (IRF3), thereby blocking the production of IFN, IFN-stimulated genes (ISGs) and antiviral cytokines. More recently, it has been reported that HBV markedly reduces IFN-β production and antiviral immunity mediated by the adapter protein, stimulator of IFN genes (STING), and to interfere with viral DNA-sensing pathways in cells [5]. The role of toll-like receptors in controlling HBV infection through innate and adaptive response has been well recognized (for a review see [6]). Thus, immunomodulatory agents that can induce innate immune responses, suppress viral replication, and additionally shape the adaptive immune response are wanted for treatment of CHB. The immunomodulatory agents, when combined with direct acting antivirals such as nucleos(t)ides can potentially result in a durable control of infection through development of neutralizing anti-HBV antibodies thereby leading to a “functional cure” of CHB within a defined duration of treatment.

SB 9200 is a small orally bioavailable prodrug of the dinucleotide SB 9000 with antiviral activity against HBV [7, 8], hepatitis C virus (HCV) [9, 10], and other RNA viruses [11]. The host immune stimulating activity of SB 9200 induces endogenous IFN *via* the activation of the viral sensor proteins, RIG-I and nucleotide-binding oligomerization domain-containing protein 2 (NOD2) [12]. Activation is believed to occur by binding of SB 9200/SB 9000 to RIG-I and NOD2 at their nucleotide binding domains. Both cytosolic proteins usually recognize signature patterns of foreign RNA such as the pathogen-associated molecular pattern (PAMP). Once PAMP is recognized, RIG-I and NOD2 become activated resulting in the induction of the IFN signaling pathway and subsequent production of type I and III IFNs, ISGs, pro-inflammatory cytokines and antiviral immune cells [13, 14]. The direct antiviral activity of SB 9200/SB 9000 is thought to inhibit the synthesis of viral nucleic acids by steric blockage of the viral polymerase, similar to the mechanism recently described for HBV [14]. The blockage, which is independent of the IFN signaling pathway, may be achieved by binding of SB 9200/SB 9000 with RIG-I and NOD2 that associate with viral RNA and that in turn prevents the polymerase from engaging with the pre-genomic (pg) RNA template for viral replication. In CHB, however, there is a need to understand whether treatment with immunomodulatory agents should be initiated in virally suppressed patients on therapy with nucleos(t)ides or in treatment-naïve patients. With these objectives in mind, the preclinical study of SB 9200 in woodchucks was initiated to assist guiding the clinical trial design of SB 9200 in CHB patients.

The Eastern woodchuck (*Marmota monax*) is naturally infected with the woodchuck hepatitis virus (WHV), a hepadnavirus which is genetically closely related to human HBV [15]. Neonatal WHV infection parallels the main route of human (vertical) transmission for chronic HBV infection and displays a disease course similar to that in HBV-infected patients. Thus, chronic WHV infection in woodchucks is a fully immunocompetent model for studying CHB and HBV-induced HCC, and woodchucks have been extensively used to evaluate efficacy and safety of current and new HBV therapeutics [15]. We recently conducted a study of SB 9200 treatment in chronic WHV carrier woodchucks at two doses of 15 and 30 mg/kg/day for 12 weeks. The study demonstrated potent, dose-dependent reductions in WHV DNA and antigens in serum and liver that were associated with (or were a result of) the induction of host antiviral immune responses [12]. In the present study, the overall antiviral response in woodchucks upon induction of innate immunity was evaluated by sequential treatment with SB 9200 followed by Entecavir (ETV) *versus* sequential treatment with ETV followed by SB 9200.

## Materials and Methods

### Investigational Drugs

SB 9200 was manufactured by Spring Bank Pharmaceuticals, Inc. (Milford, MA). The structure and antiviral characteristics of SB 9200 have been previously described [7, 16]. Doses of SB 9200 were dry mixed with woodchuck diet powder (Dyets, Bethlehem, PA) and the blended drug material suspended in ultrapure water. ETV monohydrate was obtained from AstraTech Inc. (Bristol, PA). Doses of ETV were solved in ultrapure water and mixed with woodchuck diet powder. Both drugs were orally administered to woodchucks within ½ hour after preparation.

### Sequential Treatment in Woodchucks

The animal protocol and all procedures involving woodchucks were approved by the IACUC of Georgetown University and adhered to the national guidelines of the Animal Welfare Act, the Guide for the Care and Use of Laboratory Animals, and the American Veterinary Medical Association. Woodchucks were monitored twice daily by husbandry staff of the Animal Facilities at Georgetown University for water and food intake, in addition to daily monitoring by veterinarians and/or animal personnel for well-being and general health. Assessment of physical condition was supported by weekly measurements of body weight and body temperature. Since hepatic tumors and HCC develop as a consequence of chronic WHV infection, woodchucks underwent weekly ultrasound examination of the liver. Monthly blood collections were performed for determination of hematology and clinical chemistry parameters, including liver enzymes such as gamma-glutamyl transferase (GGT), which is an oncogenic biomarker in this animal model and indicative of existing/growing liver tumors [17]. Woodchucks were anesthetized by intramuscular injection of ketamine (50 mg/kg) and xylazine (5 mg/kg). Prior to euthanasia, woodchucks were anesthetized as described above and euthanized by an overdose of Beuthanasia-D solution (80–100 mg/kg) administered by intracardiac injection, followed by bilateral intercostal thoracotomy.

**Fig 1:**
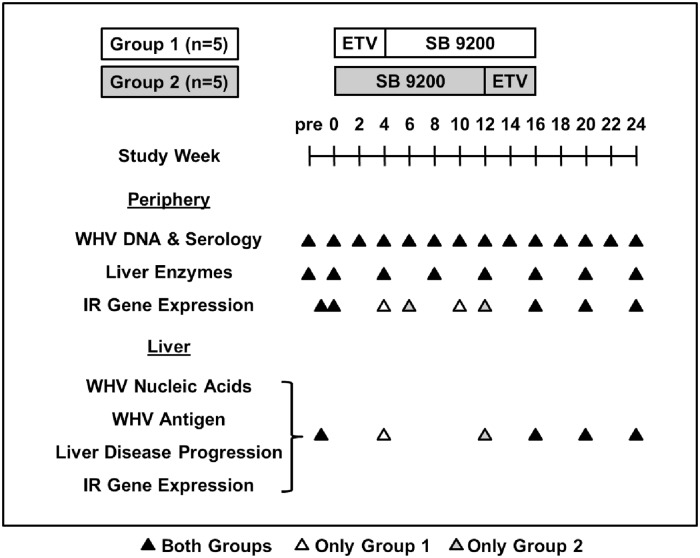
Study Design of Sequential Treatment with SB 9200 Followed by ETV or of Sequential Treatment with ETV Followed by SB 9200 in Chronic WHV Carrier Woodchucks. Two groups of 5 woodchucks each were treated once daily, orally either with SB 9200 (30 mg/kg/day) or ETV (0.5 mg/kg/day). Group 1 received ETV for 4 weeks followed by SB 9200 for 12 weeks. Group 2 received SB 9200 for 12 weeks followed by ETV for 4 weeks. WHV DNA, WHsAg and anti-WHs were determined weekly in serum. Activity of liver enzymes was determined in serum collected at pretreatment (week -2 and T_0_) and then every 4 weeks throughout the study. Host immune response (IR) gene expression was analyzed in whole blood collected at pretreatment (week -1 and T_0_), at the end of ETV treatment (week 4: Group 1; week 16: Group 2), during SB 9200 treatment (week 6: Group 2; week 10: Group 1), at the end of SB 9200 treatment (week 12: Group 2; week 16: Group 1), during follow-up (week 20), and at the end of the study (week 24). WHV nucleic acids, WHV antigens, liver disease progression markers, and host IR gene expression were determined in liver biopsy samples collected at pretreatment (week -1), at the end of ETV treatment (week 4: Group 1; week 16: Group 2), at the end of SB 9200 treatment (week 12: Group 2; week 16: Group 1), during follow-up (week 20), and at the end of the study (week 24).

Woodchucks used in this study were born in captivity and infected at 3 days of age with WHV. At the start of the study, woodchucks with chronic WHV infection were confirmed positive for serum WHV DNA and WHV surface antigen (WHsAg) and had undetectable antibodies to WHsAg (anti-WHs). Absence of liver tumors in animals with low GGT was confirmed by ultrasonography. Chronic WHV carrier woodchucks were assigned and stratified by gender, body weight, and by pretreatment serum markers (WHV DNA and WHsAg loads) and serum liver enzyme activities (GGT, sorbitol-dehydrogenase (SDH), aspartate aminotransferase (AST), and alanine aminotransferase (ALT) serum levels) into two groups (n = 5 each) (Fig 1).

Woodchucks were treated once daily, orally either with SB 9200 (30 mg/kg/day) or ETV (0.5 mg/kg/day). ETV at the selected dose was used for achieving rapid and potent suppression of WHV DNA in serum within a 4-week treatment period as also done previously in another woodchuck study [18]. Group 1 (n = 5) received ETV for 4 weeks followed by SB 9200 for 12 weeks while Group 2 (n = 5) received SB 9200 for 12 weeks followed by ETV for 4 weeks (Fig 1). As inclusion of a placebo-treated control group was not feasible due to the paucity of chronic WHV carrier woodchucks, the effects mediated by both treatment regimens were evaluated by changes in viral and host parameters from pretreatment levels which served as the control for comparison.

### Serum WHV Parameters

Serum WHV DNA concentration was determined by slot blot hybridization and samples below the limit of detection were further evaluated by PCR as described [19] (Fig 1). Serum WHsAg level and anti-WHs titer were measured by WHV-specific enzyme immunoassays as described [20] (Fig 1).

### Hepatic WHV Parameters

Hepatic levels of WHV nucleic acids were determined in liver biopsy samples as indicated in Fig 1. WHV RNA was measured by Northern blot hybridization and WHV DNA replicative intermediates (RI) and WHV covalently-closed circular (ccc) DNA were determined by Southern blot hybridization as described [19]. Liver was imunostained with antibodies against WHV core antigen (WHcAg) and WHsAg using 1:400 or 1:350 dilutions, respectively. The immunohistochemistry (IHC) scores for cytoplasmic WHcAg (cytWHcAg) and cytoplasmic or membranous WHsAg (cytWHsAg or memWHsAg) were derived from the mean of the stained hepatocyte percentage score combined with the mean of the staining intensity score. A composite IHC score of 0 indicates absence of cytWHcAg, cytWHsAg or memWHsAg in all hepatocytes (0%) whereas 8 indicates presence of strong cytWHcAg or memWHsAg staining in 81–100% or presence of strong cytWHsAg staining in 5% or more of hepatocytes. Specifically, the percentage of cytWHcAg or memWHsAg stained hepatocytes was scored on a 0–5 scale, where 0, 1, 2, 3, 4, and 5 indicate 0%, 1–20%, 21–40%, 41–60%, 61–80%, or 81–100% of cells stained, respectively. CytWHsAg was scored on a separate 0–5 scale, where 0, 1, 2, 3, 4, and 5 indicate 0%, 1%, 2%, 3%, 4%, or 5% or more of cells stained. The intensity of antigen staining was scored on a 0–3 scale, where 0, 1, 2, and 3 indicate absent, weak, moderate, or strong staining, respectively. The liver hepatitis score was derived from the mean of the lobular sinusoidal hepatitis score combined with the mean of the portal hepatitis score (n = 1–5 portal tracts examined). The composite hepatitis was scored on a 0–6 scale, where 0 = absent hepatitis, >0–2 = mild hepatitis, >2–4 = moderate hepatitis and >4 = marked to severe hepatitis. The degrees of bile duct proliferation and steatosis were scored on a 0–4 scale, where 0, 1, 2, 3 and 4 indicate absent, mild, moderate, marked, or severe disease progression.

### Host Immune Response Parameters

Immune responses associated with treatment were determined by changes in the RNA transcript levels of IFN-α, IFN-β, IFN-γ-induced protein 10 (IP-10 or CXCL10), interleukin 6 (IL-6), interferon-induced 17 kDa protein (ISG15), and 2'-5'-oligoadenylate synthetase 1 (OAS1) in blood and liver using PCR and woodchuck-specific primers and probes as described [12]. Gene expression was analyzed in whole blood and in liver as indicated in Fig 1. Woodchuck 18S rRNA expression was used to normalize target gene expression. Transcription levels of target genes were calculated as a fold-change relative to pretreatment level at week -1 (liver) or at T_0_ (blood) using the formula 2^-ΔΔ*Ct*^.

### Drug Safety Parameters

Various measurements (body weight, body temperature, clinical chemistry, and hematology) were obtained weekly to monthly for monitoring drug safety. Mortality was not observed during treatment and the remainder of the study but two woodchucks of Group 2 were euthanized during follow-up at week 20 due to the development of liver tumors.

### Statistical Analyses

All parameters were compared to the values at pretreatment and between both treatment groups using unpaired Student’s *t*-test with equal variance. *P* values of <0.05 were considered statistically significant.

## Results

### Sequential Treatment of Chronic WHV Carrier Woodchucks with SB 9200 Followed by ETV Induced Marked Suppression of Serum Viremia and Antigenemia and Delayed Recrudescence of Viral Replication Compared to Sequential Treatment with ETV Followed by SB 9200

**Fig 2:**
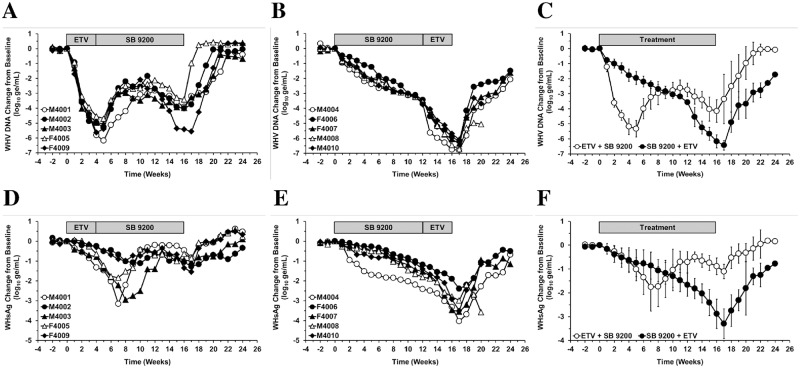
Sequential Treatment with SB 9200 Followed by ETV Induces Suppression of Serum Viremia and Antigenemia and Delays Recrudescence of Viral Replication that are Superior to Sequential Treatment with ETV Followed by SB 9200. Changes in serum WHV DNA (top panels) and WHsAg levels (bottom panels) relative to T_0_ (pretreatment baseline) in individual woodchucks administered ETV followed by SB 9200 (A, D) or SB 9200 followed by ETV (B, E), and means of each group (C, F). At T_0_, mean WHV DNA levels were 5.48x10^10^ and 8.21x10^10^ WHV genomic equivalents per mL serum and mean WHsAg levels were 2.35x10^5^ and 5.76x10^5^ ng surface protein per mL serum in Group 1 or Group 2, respectively. Error bars represent the standard error of the mean. Mean WHV DNA and WHsAg levels in Group 1 were significantly reduced compared to pretreatment during weeks 1–19 or weeks 5–12 and weeks 14–18, respectively (all *p*<0.05). In Group 2, mean WHV DNA and WHsAg levels were significantly reduced from pretreatment during weeks 1–24 or weeks 2–24, respectively (all *p*<0.05).

Sequential treatment with ETV followed by SB 9200 (Group 1) or with SB 9200 followed by ETV (Group 2) for a total of 16 weeks was well tolerated, and no signs of overt toxicity were observed based on gross observations, body weights, body temperatures, hematology, and clinical chemistry (data not shown). In Group 1, ETV treatment induced fast declines in serum WHV DNA in all woodchucks, and the average reduction at week 4 was 5.2 log_10_ while the maximum average reduction at week 5 was 5.3 log_10_ from pretreatment (Fig 2A–2C).

Serum WHsAg also declined during ETV treatment in all woodchucks, with an average reduction of 0.7 log_10_ at week 4 and of 1.0 log_10_ at week 5 from pretreatment (Fig 2D–2F), but reductions were less pronounced and more variable in individual animals when compared to serum WHV DNA. After switching to SB 9200 treatment, WHV DNA increased within the initial 7 weeks but stayed below baseline; thereafter, viremia declined again and the average reduction at the end of SB 9200 treatment was 4.2 log_10_ compared to pretreatment. A comparable pattern was observed for serum WHsAg in this group, with an average reduction in antigenemia of 1.1 log_10_ from pretreatment one week after the end of sequential treatment. Of note is that the kinetic of viremia and antigenemia declines following the treatment switch from ETV to SB 9200 differed in three animals. While serum WHV DNA in woodchucks M4001, M4003 and F4005 increased similar to the other two animals of this group (M4002 and F4009), serum WHsAg declined further for additional 2–3 weeks before a rebound was observed. Although the effect on WHsAg was not sustained, this apparent disconnection in the kinetics of viremia and antigenemia is unclear at the moment but could suggest an added overlapping treatment effect of SB 9200 on this viral antigen. In Group 2, SB 9200 treatment induced gradual and rather uniform declines in serum WHV DNA and WHsAg in all woodchucks, except for M4004 in which the decline in antigenemia was more pronounced (Fig 2B–2E). At the end of SB 9200 treatment, the average reduction in WHV DNA and WHsAg was 3.6 log_10_ or 1.7 log_10_, respectively, from pretreatment (Fig 2C–2F). After switching to ETV treatment, viremia and antigenemia declined further and the average reduction observed one week after the end of treatment was 6.4 log_10_ or 3.3 log_10_, respectively, compared to pretreatment. After cessation of treatment, immediate rebound in viremia and antigenemia was observed in Group 1, and WHV DNA and WHsAg returned to pretreatment within 2–6 or 3–8 weeks, respectively, indicating some variability among individual woodchucks. Recrudescence of viral replication in Group 2 was significantly delayed as viremia and antigenemia in all woodchucks never returned to pretreatment within the 8-week follow-up period and WHV DNA and WHsAg stayed 1.7 log_10_ or 0.8 log_10_, respectively, below baseline at the end of the study. It is of note that serum WHsAg levels of four woodchucks of this group were near the lower limit of quantification of the ELISA assay (M4004, F4007 and M4010 at week 17 and M4008 at week 20), suggesting that continued sequential treatment beyond 16 weeks may result in much greater suppression of WHV viremia and antigenemia, including undetectable WHsAg. Overall, mean serum WHV DNA of Group 1 was significantly lower than in Group 2 during weeks 1–7 (all *p*<0.05). In Group 2, mean WHV DNA and WHsAg were significantly lower than in Group 1 during weeks 12–24 or weeks 13–19 and weeks 21–23, respectively (all *p*<0.05). As the treatment regimens at the doses and duration applied in Groups 1 and 2 were unable to produce complete and durable loss of detectable WHsAg (and WHV DNA) in woodchucks, seroconversion to anti-WHs antibodies was not observed (data not shown).

### Treatment with SB 9200 Followed by ETV Resulted in more Pronounced Reduction in Hepatic Levels of WHV Nucleic Acids than Treatment with ETV Followed by SB 9200

**Fig 3:**
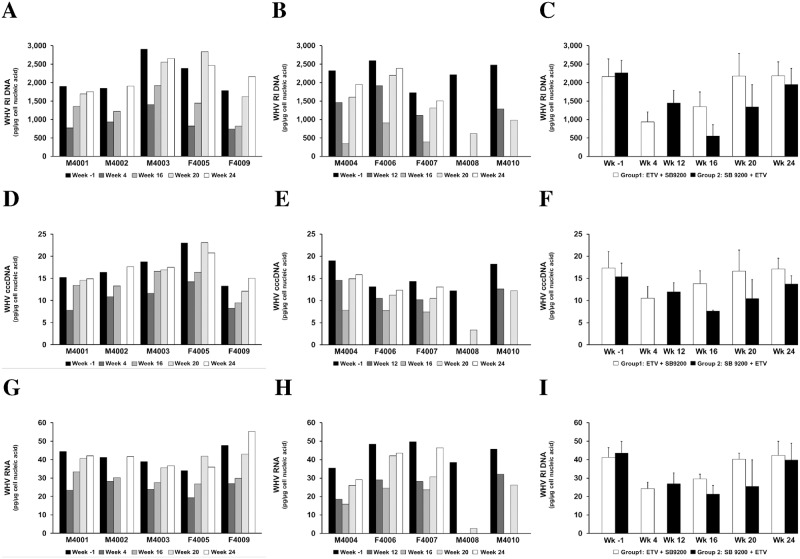
Sequential Treatment with SB 9200 Followed by ETV Results in more Pronounced Reduction in Hepatic Levels of WHV Nucleic Acids than Sequential Treatment with ETV Followed by SB 9200. Changes throughout the study in hepatic levels of WHV RI DNA (top panels), WHV cccDNA (middle panels) and WHV RNA (bottom panels) in individual woodchucks treated with ETV followed by SB 9200 (A, D, G) or treated with SB 9200 followed by ETV (B, E, H) and mean of each group (C, F, I). At week -1, mean levels for WHV RI DNA, WHV cccDNA, and WHV RNA were 2162.3 and 2267.4 pg/μg total DNA, 17.3 and 15.4 pg/μg total DNA, and 41.2 and 43.6 pg/μg total RNA in Group 1 or Group 2, respectively. Error bars represent the standard error of the mean. Mean WHV RI DNA, WHV cccDNA, and WHV RNA levels in Group 1 were significantly reduced compared to pretreatment at weeks 4 and 16, at week 4, or at weeks 4 and 16, respectively (all *p*<0.05). In Group 2, mean WHV RI DNA, WHV cccDNA and WHV RNA levels were significantly reduced from pretreatment at weeks 12, 16 and 20, at week 16, or at weeks 12, 16 and 20, respectively (all *p*<0.05).

Both treatment regimens induced marked reductions in the levels of hepatic WHV RNA, WHV cccDNA, and WHV RI DNA when compared to pretreatment (Fig 3). Although liver biopsies could not be collected from all woodchucks of Group 2 at the end of treatment, the declines in these viral markers correlated well with the reductions in serum viremia and antigenemia (compare Figs 2 and 3).

The average reduction of WHV RI DNA, WHV cccDNA, and WHV RNA in Group 1 after the end of ETV treatment was 0.37 log_10_, 0.22 log_10_ and 0.23 log_10_ from pretreatment. Due to the rapid recrudescence of WHV replication following the stop of ETV dosing and switching to SB 9200 treatment, the average decline in WHV RI DNA, WHV cccDNA, and WHV RNA at the end of the 16-week treatment period was only 0.21 log_10_, 0.10 log_10_ and 0.14 log_10_ from baseline in this group. In Group 2, average reduction of WHV RI DNA, WHV cccDNA, and WHV RNA after the end of SB 9200 treatment was 0.20 log_10_, 0.13 log_10_ and 0.23 log_10_ from pretreatment. After switching to ETV treatment WHV nucleic acid levels declined further, with an average reduction in WHV RI DNA, WHV cccDNA, and WHV RNA of 0.64 log_10_, 0.30 log_10_ and 0.32 log_10_ from baseline at the end of the 16-week treatment period. The overall data indicate that the antiviral effect of sequential treatment with ETV followed by SB 9200 or with SB 9200 followed by ETV was most pronounced for WHV RI DNA. After the end of treatment, rebound of WHV nucleic acids was observed in Group 1, and levels returned to pretreatment within 4–8 weeks. Rebound of WHV nucleic acids was delayed in Group 2 and levels never returned to pretreatment as WHV RI DNA, WHV cccDNA, and WHV RNA stayed 0.06 log_10_, 0.05 log_10_ and 0.05 log_10_ below baseline at the end of the study. Overall, mean WHV RI DNA, WHV cccDNA and WHV RNA of Group 2 were significantly lower than in Group 1 at week 16 (all *p*<0.05).

### Treatment with SB 9200 Followed by ETV Resulted in Greater Reduction of Hepatic WHV Antigen Expression than Treatment with ETV and SB 9200

**Fig 4:**
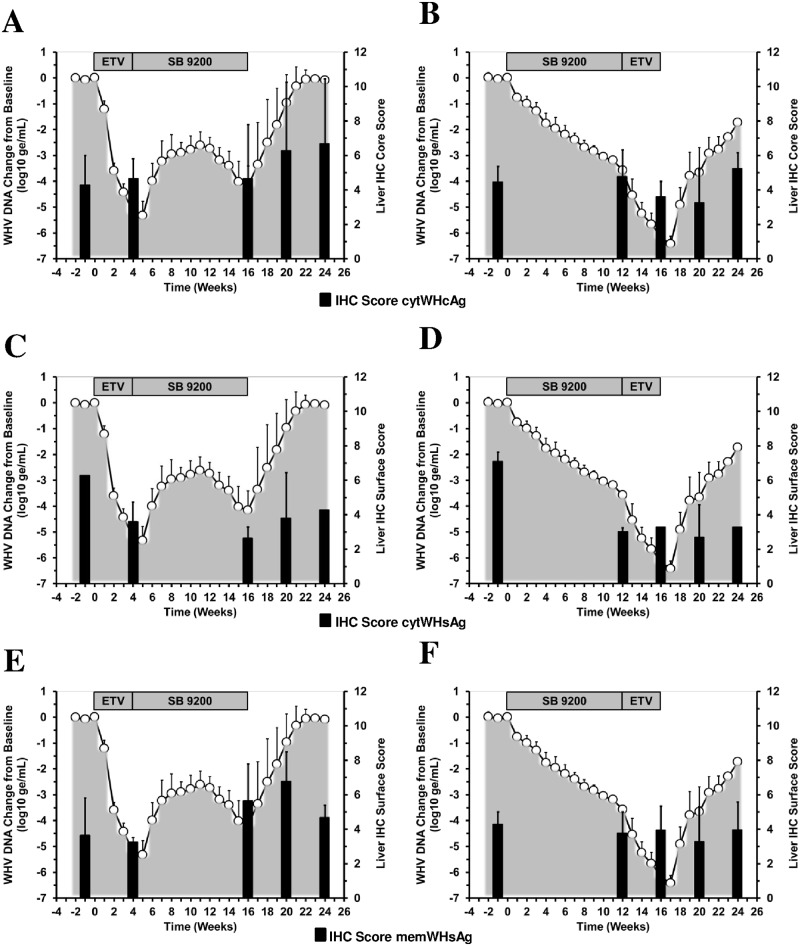
Sequential Treatment with SB 9200 Followed by ETV Results in Greater Reduction of Hepatic WHV Antigen Expression than Sequential Treatment with ETV and SB 9200. Changes in mean IHC scores for cytWHcAg (top panels), cytWHsAg (middle panels), and memWHsAg expression (bottom panels) in response to treatment with ETV followed by SB 9200 (A, C, E) or SB 9200 followed by ETV (B, D, F) Changes in mean serum WHV DNA relative to T_0_ (pretreatment baseline) is plotted on the left y-axis. The mean IHC scores for antigen expression in liver are plotted on the right y-axis. Error bars represent the standard error of the mean. Mean IHC scores for cytWHcAg and memWHsAg in Group 1 were significantly higher compared to pretreatment at weeks 20 and 24 or at week 20, respectively (all *p*<0.05). Mean IHC scores for cytWHsAg in Groups 1 and 2 were significantly lower compared to pretreatment at weeks 4, 16, 20, and 24 or at weeks 12, 16, 20, and 24, respectively (all *p*<0.05).

Both treatment regimens caused transient reductions in hepatic expression of cytWHcAg, cytWHsAg, and memWHsAg from pretreatment level that were overall more pronounced and durable in Group 2 than in Group 1 (Fig 4).

At the end of the 16-week treatment period, minor reductions for cytWHcAg and memWHsAg were only observed in woodchucks treated with SB 9200 followed by ETV but not in animals treated with ETV followed by SB 9200. Both treatment regimens, however, induced pronounced declines in cytWHsAg after 16 weeks. After cessation of treatment, cytWHcAg and memWHsAg in Group 1 increased further while cytWHsAg started to rebound. The average IHC scores for cytWHcAg and memWHsAg at the end of the study in this group were higher than those observed at pretreatment (the increase was more pronounced for cytWHcAg than memWHsAg) but stayed below baseline for cytWHsAg. Following the end of treatment in Group 2, antigen expression declined further during follow-up. At the end of the study, the average IHC scores in this group were higher (cytWHcAg) or lower (memWHsAg and cytWHsAg) than those seen at pretreatment. Overall, the mean IHC scores for cytWHcAg and memWHsAg in Group 2 were significantly lower than in Group 1 at week 20 (both *p*<0.05). Contrary, the mean IHC score for cytWHsAg of Group 2 was significantly higher than in Group 1 at week 16 *(p*<0.05).

### Treatment with SB 9200 Followed by ETV or Treatment with ETV Followed by SB 9200 Slowed Down Liver Disease Progression

**Fig 5:**
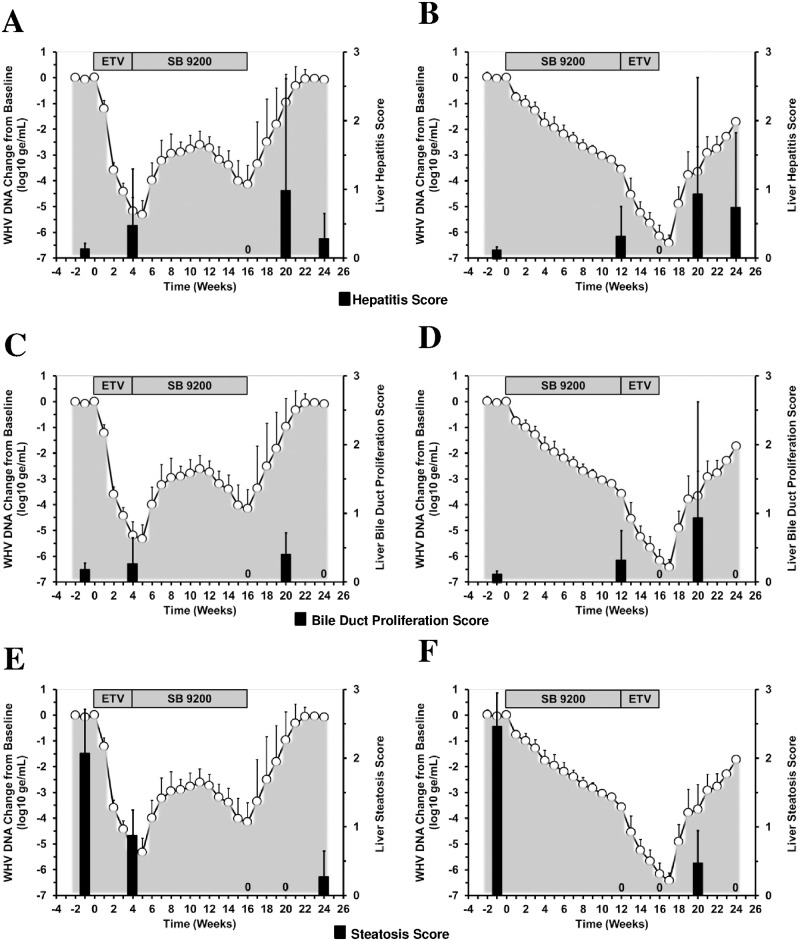
Sequential Treatment with SB 9200 Followed by ETV or Sequential Treatment with ETV Followed by SB 9200 Slows Down Liver Disease Progression. Changes in mean scores for portal and lobular sinusoidal hepatitis (top panels), bile duct proliferation (middle panels), and steatosis (bottom panels) in response to treatment with ETV followed by SB 9200 (A, C, E) or SB 9200 followed by ETV (B, D, F). Changes in mean serum WHV DNA relative to T_0_ (pretreatment baseline) is plotted on the left y-axis. The mean scores for liver disease parameters are plotted on the right y-axis. Error bars represent the standard error of the mean. Mean scores for steatosis in Groups 1 and 2 were significantly lower compared to pretreatment at weeks 4, 16, 20, and 24 or at weeks 12, 16, 20, and 24, respectively (all *p*<0.05). Statistically significant changes from pretreatment were not observed for the mean scores for portal and lobular sinusoidal hepatitis and for bile duct proliferation.

Both treatment regimens further correlated temporally with undetectable liver inflammation, bile duct proliferation, and steatosis in all woodchucks at the end of the 16-week treatment period (Fig 5). After cessation of treatment, the composite scores for lobular sinusoidal and portal hepatitis increased in 1–2 woodchucks of both groups during follow-up but declined again at the end of the study.

A comparable pattern was observed for bile duct proliferation after the end of treatment which increased in the same animals across both groups during follow-up but was undetectable at the end of the study. After the cessation of treatment, steatosis in woodchucks of Group 1 stayed undetectable during follow-up but increased in one animal at the end of the study. In Group 2, steatosis transiently increased after the end of treatment in two woodchucks during follow-up and was again undetectable at the end of the study. The trend to undetectable disease markers at the end of treatment and to reduced liver inflammation, undetectable bile duct proliferation, and minimal (Group 1) or undetectable steatosis (Group 2) at the end of the study was comparable, with no statistically significant differences between the treatment regimens (*p*>0.05).

**Fig 6:**
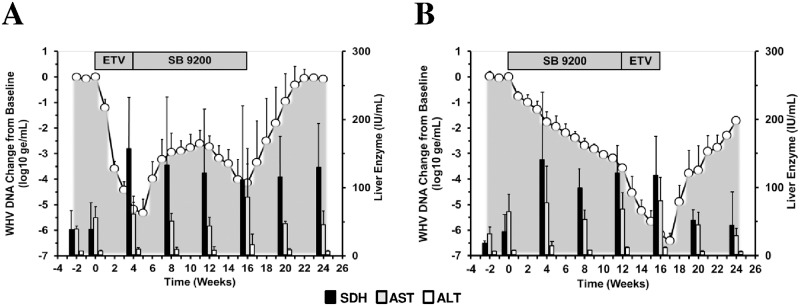
Sequential Treatment with SB 9200 Followed by ETV or Sequential Treatment with ETV Followed by SB 9200 is Associated with Elevated Serum Activity of Liver Enzymes. Changes in mean serum levels of SDH, AST and ALT in response to treatment with ETV followed by SB 9200 (A) or SB 9200 followed by ETV (B). Changes in mean serum WHV DNA relative to T_0_ (pretreatment baseline) is plotted on the left y-axis. The mean serum levels of liver enzymes are plotted on the right y-axis. Error bars represent the standard error of the mean. Mean serum levels for SDH in Groups 1 and 2 were significantly higher compared to T_0_ (pretreatment baseline) at weeks 4, 20 and 24 or at weeks 8, 12 and 16, respectively (all *p*<0.05). Mean serum level of AST in Group 2 was significantly lower compared to T_0_ at week -2 (*p*<0.05). Mean serum level of ALT in Group 2 was significantly higher compared to week T_0_ at week 16 (*p*<0.05).

Serum activities of the liver enzymes SDH, AST and ALT are routinely used in chronic WHV carrier woodchucks for the biochemical assessment of hepatic injury possibly caused by antiviral treatment [17]. There was a trend towards elevated serum SDH level in Group 1, especially at the end of ETV treatment, and serum activity of this liver enzyme stayed rather high until the end of the study (Fig 6). A transient increase in the serum levels of AST and ALT was observed at the end of SB 9200 treatment although the elevation was less pronounced for ALT.

In Group 2, all three liver enzymes were increased after 4 weeks of SB 9200 treatment although the rise in ALT was less remarkable. During the remainder of SB 9200 treatment, serum levels of SDH and AST stayed elevated and continued to increase until the end of ETV treatment; thereafter serum activity of these liver enzymes declined during follow-up despite the beginning recrudescence of viral replication and became normalized at the end of the study. On a group level, however, these differences were not statistically significant between the treatment regimens (*p*>0.05). Overall, there was a temporal association between antiviral response and elevated serum activity of SDH and AST, especially in Group 2, that may be indicative of the host immune response induced by sequential treatment with SB 9200 and ETV. These observations are supported by the induction of IFNs and ISGs in treated woodchucks (see below).

### Treatment with SB 9200 Followed by ETV Induced Pronounced Expression Increases of Type I IFNs, Cytokine and ISGs in Blood

**Fig 7:**
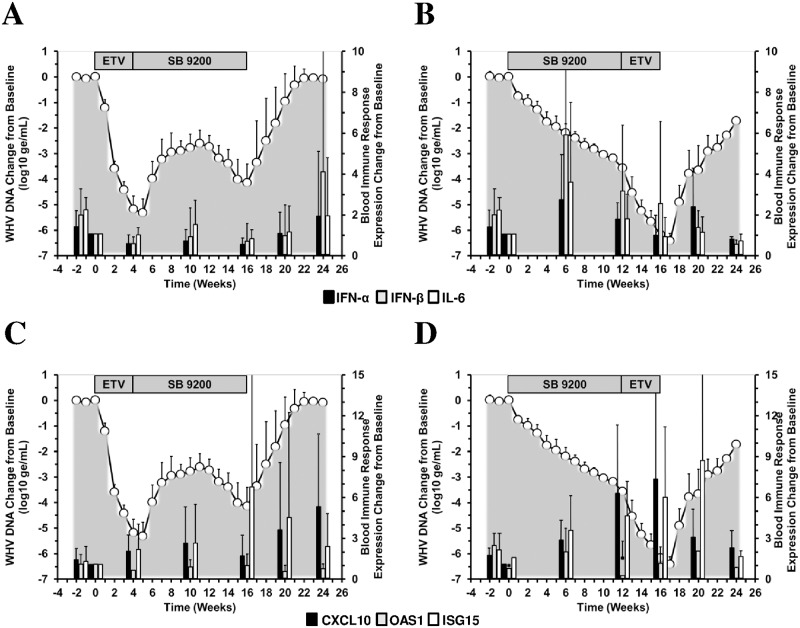
Sequential Treatment with SB 9200 Followed by ETV Induces Pronounced Expression Increases of Type I IFNs, Cytokine and ISGs in Blood. Changes in mean blood transcript levels of IFN-α, IFN-β, and IL-6 (top panels) and of CXCL10, OAS1 and ISG15 (bottom panels) in response to treatment with ETV followed by SB 9200 (A, C) or SB 9200 followed by ETV (B, D). Changes in mean serum WHV DNA relative to T_0_ (pretreatment baseline) is plotted on the left y-axis. The mean blood transcript levels are plotted on the right y-axis. Error bars represent the standard error of the mean. Mean transcript levels for IFN-α in Groups 1 and 2 were significantly lower compared to T_0_ (pretreatment baseline) at weeks 16 or 24, respectively (both *p*<0.05). Mean transcript levels for IFN-β and OAS1 in Groups 1 and 2 were significantly lower at weeks 4 or 24, respectively (all *p*<0.05). Mean transcript level for IL-6 in Group 1 was significantly higher at week -2 (*p*<0.05). Mean transcript level for CXCL10 in Group 2 was significantly higher at week 6 (*p*<0.05). Mean transcript level for ISG15 in Group 2 was significantly higher at weeks 12 and 24 (both *p*<0.05).

Sequential treatment with SB 9200 followed by ETV transiently induced the mRNA expression of type I IFNs (i.e., IFN-α and IFN-β) and the pro-inflammatory cytokine, IL-6, in blood when compared to pretreatment, at higher transcript levels than sequential treatment with ETV followed by SB 9200 (Fig 7). Both treatment regimens also induced the expression of select antiviral ISGs (i.e., mainly CXCL10 and ISG15 but not OAS1) from pretreatment in blood, with a marked induction of ISG15 in both groups at the end of the 16-week treatment period (Fig 7).

Induction of ISG15 was associated with transiently increased expression of CXCL10 in Group 2 but not in Group 1. While ISG15 expression was transient in Group 1 after cessation of treatment, it increased further in Group 2 during follow-up but then declined at the end of the study. Conversely, expression of CXCL10 in Group 1 increased during follow-up until the end of the study. On a group level, however, these overall differences were not statistically significant between the treatment regimens (*p*>0.05). Considering that ETV treatment in Group 1 failed to induce marked expression of the above genes in blood, and that SB 9200 treatment in Group 2 was associated with suppression of WHV replication, these results suggest that the induction of host innate immunity plays a crucial role in the antiviral response mediated by SB 9200. Furthermore, the more pronounced immune response observed during sequential treatment with SB 9200 followed by ETV apparently contributed to the superior antiviral effect of this treatment regimen.

### Delayed Recrudescence of Viral Replication After Treatment with SB 9200 Followed by ETV was Associated with Expression Increases of Type I IFNs, Cytokine and ISGs in Liver

**Fig 8:**
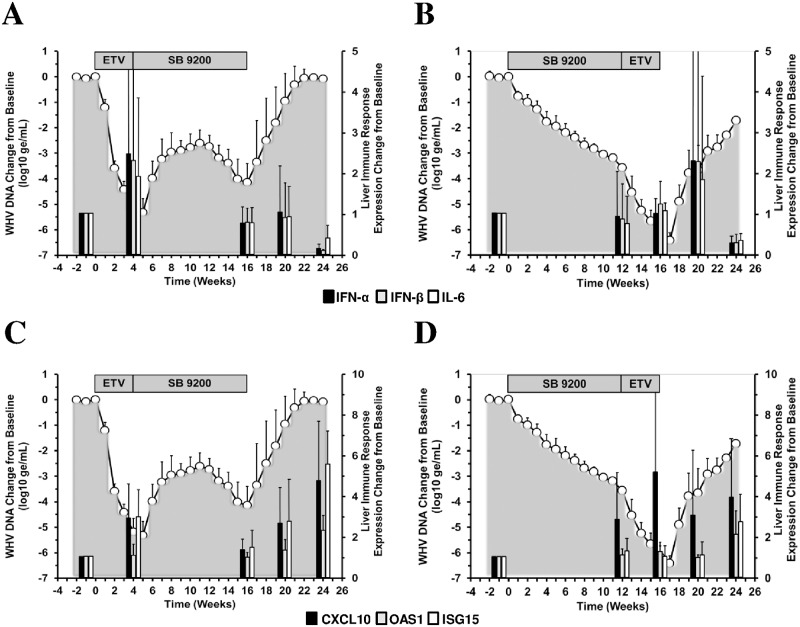
Delayed Recrudescence of Viral Replication after Sequential Treatment with SB 9200 Followed by ETV is Associated with Expression Increases of Type I IFNs, Cytokine and ISGs in Liver. Changes in mean liver transcript levels of IFN-α, IFN-β, and IL-6 (top panels) and of CXCL10, OAS1 and ISG15 (bottom panels) in response to treatment with ETV followed by SB 9200 (A, C) or SB 9200 followed by ETV (B, D). Changes in mean serum WHV DNA relative to T_0_ (pretreatment baseline) is plotted on the left y-axis. The mean liver transcript levels are plotted on the right y-axis. Error bars represent the standard error of the mean. Mean transcript levels for IFN-α, IFN-β and IL-6 in Groups 1 and 2 were significantly lower compared to week -1 (pretreatment baseline) at week 24 (all *p*<0.05). Mean transcript levels for CXCL10 and ISG15 in Group 1 were significantly higher at weeks 4 and 24 (all *p*<0.05). Mean transcript level for ISG15 in Group 2 was significantly higher at week 24 (*p*<0.05). Mean transcript level for OAS1 in Group 1 was significantly higher at week 24 (*p*<0.05).

Both treatment regimens also induced mRNA expression of type I IFNs, cytokine and ISGs in liver although with temporal differences (Fig 8).

Contrary to the observations in the periphery, treatment with ETV in Group 1 induced transient expression of IFN-α, IFN-β, IL-6, CXCL10, and ISG15 in liver. At the end of SB 9200 treatment in this group, transcript levels of immune response genes were reduced but increased expression of ISGs, including OAS1 that was not observed in the periphery, was noted during follow-up until the end of the study. Different to the expression of cytokines in blood which was observed during SB 9200 treatment in Group 2, IFN-α, IFN-β and IL-6 in liver transiently increased at the end of ETV treatment, with a peak during follow-up and a decline at the end of the study. Comparable to the periphery, CXCL10 expression in Group 2 increased at the end of SB 9200 treatment, and even more so at the end of the 16-week treatment period and stayed elevated thereafter. Increased expression of ISG15 and OAS1 was also seen at the end of the study. Despite these deviations, hepatic gene expression was comparable between both treatment regimens (*p*>0.05). Since hepatic expression of innate immune response genes in Group 2 lasted beyond the end of treatment, and peak expression of most genes was observed between follow-up and the end of the study, these results suggest that the delayed recrudescence of viral replication after sequential treatment with SB 9200 followed by ETV was (in part) a consequence of the induced host innate immunity. As viral relapse was eventually observed, the results further indicate that additional antiviral immune mechanisms contributed to the overall treatment response to SB 9200 and ETV. As mentioned above, a longer duration of treatment with SB 9200 may have resulted in more sustained virological response.

## Discussion

For evaluating the overall antiviral response against HBV, chronic WHV carrier woodchucks were first treated with SB 9200 for induction of innate immune response followed by treatment with ETV for additional reduction of viral burden. The efficacy, safety, and pharmacodynamics associated with this treatment regimen were compared to treatment, first with ETV for initial rapid suppression of WHV replication followed by immune modulation with SB 9200. Induction of host immunity by pretreatment with SB 9200 followed by ETV resulted in multi-log reductions in serum viremia and antigenemia (Fig 2) and in noticeable declines in hepatic WHV nucleic acids (Fig 3) and antigens (Fig 4) as well as slowed liver disease progression (Fig 5) and delayed viral relapse following treatment discontinuation, all of which was considered superior to the antiviral response observed when using the strategy of viral reduction with ETV followed by immune modulation with SB 9200.

During infections, viral RNA is mainly sensed by pattern-recognition receptors such as RIG-1 and NOD2 [21, 22]. Binding of these sensor proteins to PAMP within the viral RNA activates downstream signaling pathways leading to the induction of type I and type III IFNs and inflammatory cytokines [13, 23]. Thus, sensing of viral RNA is a crucial process to induce antiviral innate immune responses for limiting viral replication and for activation of adaptive immunity [23]. SB 9200 has potent antiviral activity against wild-type and drug-resistant variants of HBV [7, 8], and also against WHV [12]. In analogy to the mechanism described for HBV [14], it appears that the direct antiviral property of SB 9200 may involve interference of the hepadnavirus polymerase to engage with the HBV or WHV pgRNA by SB 9200 activated RIG-I and NOD2. The assumption is consistent with the antiviral efficacy of SB 9200 in HBV transgenic mice, an inherently immunotolerant animal model of chronic HBV infection, in which monotherapy with SB 9200 resulted in significant reduction in liver HBV DNA that was comparable to that of Adefovir [8]. Furthermore, SB 9200 has additionally antiviral activity against RNA viruses, including, HCV, Norovirus and Respiratory Syncytial Virus [9–11], consistent with the expectation that activation of viral sensors by this compound should be independent of the type of virus and genotypes. In woodchucks, monotherapy with SB 9200 for 12 weeks induced dose-dependent effects on WHV replication which were associated with the expression of IFN-α, IFN-β and ISGs in blood and liver, indicating that the host immune stimulating property of SB 9200 apparently is an important contributor to the overall antiviral activity of this compound [12]. Since host innate immune responses in the previous study correlated with a prolonged activation of the RIG-I/NOD2 pathway, including upregulated expression of RIG-I, NOD2, STING, and IRF3, and presence of elevated RIG-I protein in liver, this suggested that SB 9200 induces endogenous type I IFNs *via* the activation of viral sensor proteins. As dose-dependent recrudescence of WHV replication following discontinuation of monotherapy with SB 9200 was observed [12], the present study tested the antiviral effects of SB 9200 during sequential treatment with ETV. The applied treatment regimen to woodchucks, involving SB 9200 in combination with a nucleos(t)ide, is considered the most likely therapeutic option of this compound for future treatment of HBV in patients.

The overall antiviral response induced by sequential treatment with SB 9200 followed by ETV in the present study was in the range of those of nucleos(t)ides previously evaluated in woodchucks. The 6.4 log_10_ magnitude of viral load reduction in serum observed in Group 2 after 16 weeks of sequential treatment (Fig 2) was superior to monotherapy with Lamivudine, Emtricitabine, Tenofovir, and Adefovir after administration for 12 to 16 weeks [19] but comparable to ETV and Clevudine given for a similar duration [24, 25]. As SB 9200 has immune stimulating activity, in addition to its direct antiviral property, the maximum reduction in serum viremia was also in the range obtained with immunomodulators recently tested in woodchucks, including recombinant woodchuck IFN-α5 (only in 3 of 7 (43%) woodchucks responding to treatment), GS-9620 (a toll-like receptor 7 agonist), and ETV in combination with an antibody to programmed cell death-1 ligand 1 (anti-PD-L1) [25–27]. In contrast to monotherapy with Lamivudine, Emtricitabine, Tenofovir, and Adefovir but comparable to Clevudine, ETV alone or in combination with anti-PD-L1, and IFN-α5 in responder woodchucks, sequential treatment with SB 9200 followed by ETV resulted in marked WHsAg reduction of 3.3 log_10_ (Fig 2). Common for treatment with SB 9200 followed by ETV and for most other compounds and treatment regimens described above, except for GS-9620, ETV plus anti-PD-L1, and responder woodchucks to IFN-α5 treatment, was the viral rebound following treatment discontinuation. Furthermore, and comparable to most other compounds and combination treatment strategies (but not for GS-9620 and ETV plus anti-PD-L1), seroconversion was not observed as SB 9200 followed by ETV did not produce (sustained) loss of WHsAg.

Comparable to sequential treatment with SB 9200 followed by ETV (Fig 6), the above compounds and treatment regimens also induced transient increases in liver enzymes in woodchucks during treatment, before serum activity of SDH and AST (and ALT) became normalized at the end of treatment. Since the SDH level in Group 2 peaked at week 4, stayed elevated until the end of the 16-week treatment period, and was temporally associated with initial and then marked reductions in serum WHsAg, hepatic WHV cccDNA, and hepatic WHcAg and WHsAg expression, the rise in this liver enzyme and its durable presence thereafter may indicate in part immune-mediated viral clearance of infected hepatocytes by cytotoxic effector cells as it has been also suggested in other treatment studies in woodchucks with immunomodulators such as IFN-α5 and GS-9620 [26, 27]. However, as liver inflammation was undetectable at the end of treatment (Fig 5), this may further indicate that other, non-cytolytic mechanism(s) contributed to the peak suppression of WHV replication as also observed during treatment with IFN-α5 and GS-9620 [26, 27]. General cytotoxicity or even adverse effects mediated by the applied treatment regimens were not noted, and the elevated serum transaminase activity was not associated with necrosis in liver (data not shown). Interestingly, the SDH level in Group 1 also peaked at week 4 at the end of ETV treatment and then stayed elevated through the SB 9200 treatment period until the end of the study but this was not associated with a comparable reduction of WHV markers in serum and liver as observed in Group 2. The rise in serum transaminase activity in this group, therefore, may indicate a different underlying cause such as mediated by a pro-inflammatory immune response rather than by cytotoxic effector cells (see below).

Considering the diminished or impaired innate immune response in chronic HBV and WHV infections [28–31], the observed peripheral and hepatic induction of IFN-α, IFN-β and ISGs such as CXCL10 and ISG15, and the pro-inflammatory cytokine, IL-6, during sequential treatment with SB 9200 followed by ETV is important because it suggests that an antiviral innate immune response was induced (Figs 7 and 8). Since the host immunity induced in Group 2 lasted even beyond treatment, it apparently also modulated the viral rebound following treatment discontinuation. This is further supported by the observation that the immediate recrudescence of viral replication in Group 1 was not temporally associated with innate immune responses in blood and liver. Since recrudescence of viral replication following cessation of prolonged monotherapy with ETV can be variable in individual woodchucks [32, 33], it could be argued that viral relapse from ETV treatment is itself slow, or slower than relapse from SB 9200 treatment, thereby causing the delayed rebound that was observed in Group 2. While the inclusion of control groups receiving ETV and SB 9200 alone in the present study was not possible for addressing this question, a comparison of viral rebound kinetics following 4 weeks of monotherapy with ETV [18, 33] or 12 weeks of monotherapy with SB 9200 [12] using the same doses as applied in the present study indicates that viral relapse occurs rather rapidly with either drug and that rebound from ETV treatment is not markedly slower than from SB 9200 treatment.

Considering the above result on long-lasting host immunity in Group 2, and the transient rebound of serum viremia and antigenemia in Group 1 during SB 9200 treatment after discontinuation of ETV dosing, it appears that SB 9200 needs to be administered for a certain duration before it unfolds its full immune stimulating potential. This assumption is supported by the results of the previous study in woodchucks [12] that suggest activation of the viral sensor pathway and induction of host immune response after 6–12 weeks of SB 9200 administration. Once induced by SB 9200, activation of the RIG-I/NOD2 pathway and induction of host immunity is present in woodchucks for a prolonged time. Additionally, the elevated intrahepatic levels of IFN-α, IFN-β, CXCL10, and ISG15 in Group 1 at week 4 (Fig 8) may indicate that host innate immune response was preactivated to some extent at the end of ETV treatment. Together with the increased IL-6 level in liver (Fig 8), indicating the presence of a pro-inflammatory immune response, and the rise in serum SDH activity at this time (Fig 6), this overall suggests that the hepatic immune environment in Group 1 was apparently different at the start of SB 9200 treatment when compared to Group 2. Since treatment with SB 9200 aims at inducing an endogenous type I IFN response *via* the activation of the RIG-I/NOD2 pathway, it appears that the applied immunomodulatory therapy is more efficient when innate and/or pro-inflammatory immune responses are not already activated in liver as it was the situation in Group 2. Differences in the antiviral response to IFN-α treatment that are determined by preactivation of host immune responses have been described in HCV-infected patients [34–36].

An interesting finding of the present and previous studies in woodchucks was that the antiviral response to SB 9200, alone or followed by ETV, did not correlate well with the long-lasting hepatic expression of antiviral ISGs tested, suggesting that other immune response and/or antiviral mechanisms may play a role, especially in the peak response to treatment. This assumption is plausible considering that humans, chimpanzees, and woodchucks resolve HBV and WHV infection without inducing a strong type I IFN response [29, 31, 37]. However, there are several limitations to consider: Since only three antiviral ISGs were tested, peripheral and hepatic expression of other antiviral ISGs may be important to and correlate with treatment response. In addition, as the collection of liver biopsy materials was restricted to the begin and end of the 12-week treatment period with SB 9200, significant induction of type I IFNs and most ISGs by this compound in liver during treatment may have been missed. Furthermore, the durable expression of antiviral ISGs beyond the end of sequential treatment may not entirely attributable to SB 9200 and ETV and could additionally include an immune response of the host to the recurrence of viral replication following cessation of treatment. Since gene expression during treatment was restricted to 2–4 hours post-dose, it is likely that maximum expression of immune response genes was not detected and that peak induction may be associated with the antiviral response to SB 9200.

## Conclusions

In summary, the induction of host innate immune response in woodchucks by pretreatment with SB 9200 followed by ETV administration for additional viral load reduction resulted in declines in WHV DNA, WHV RNA, and WHV proteins in blood and liver. The magnitude of suppression in WHV replication during sequential treatment and the delayed viral rebound upon treatment discontinuation were superior to the antiviral effect obtained by using the strategy of viral load reduction with ETV followed by immune modulation with SB 9200. Antiviral efficacy of SB 9200 during the latter treatment regimen may have been limited by the innate and/or pro-inflammatory immune responses already present in liver. Considering all results, it appears that an immunotherapy designed at modulating the endogenous IFN-α/IFN-β response is more likely to be successful in a liver environment that is characterized by absent or only limited preactivation of host immune responses. These data in woodchucks, a fully immunocompetent animal model of CHB, support the ongoing Phase II clinical trials of SB 9200, alone as well as sequential, in combination studies with a nucleoside, in the treatment of chronic HBV in patients.

## References

[1] Lavanchy D. Hepatitis B virus epidemiology, disease burden, treatment, and current and emerging prevention and control measures. J Viral Hepat. 2004;11(2):97–107. 14996343 10.1046/j.1365-2893.2003.00487.x

[2] Lee JY, Locarnini S. Hepatitis B virus: pathogenesis, viral intermediates, and viral replication. Clin Liver Dis. 2004;8(2):301–20. 15481342 10.1016/j.cld.2004.02.009

[3] Schweitzer A, Horn J, Mikolajczyk RT, Krause G, Ott JJ. Estimations of worldwide prevalence of chronic hepatitis B virus infection: a systematic review of data published between 1965 and 2013. Lancet. 2015;386(10003):1546–55. 10.1016/S0140-6736(15)61412-X 26231459

[4] Kwon H, Lok AS. Hepatitis B therapy. Nat Rev Gastroenterol Hepatol. 2011;8(5):275–84. 10.1038/nrgastro.2011.33 21423260

[5] Liu Y, Li J, Chen J, Li Y, Wang W, Du X, et al Hepatitis B virus polymerase disrupts K63-linked ubiquitination of STING to block innate cytosolic DNA-sensing pathways. J Virol. 2015;89(4):2287–300. 10.1128/JVI.02760-14 25505063 PMC4338878

[6] Ma Z, Zhang E, Yang D, Lu M. Contribution of Toll-like receptors to the control of hepatitis B virus infection by initiating antiviral innate responses and promoting specific adaptive immune responses. Cell Mol Immunol. 2015;12(3):273–82. 10.1038/cmi.2014.112 25418467 PMC4654312

[7] Iyer RP, Padmanabhan S, Zhang G, Morrey JD, Korba BE. Nucleotide analogs as novel anti-hepatitis B virus agents. Curr Opin Pharmacol. 2005;5(5):520–8. 10.1016/j.coph.2005.04.019 16087397

[8] Iyer RP, Roland A, Jin Y, Mounir S, Korba B, Julander JG, et al Anti-hepatitis B virus activity of ORI-9020, a novel phosphorothioate dinucleotide, in a transgenic mouse model. Antimicrob Agents Chemother. 2004;48(6):2318–20. 10.1128/AAC.48.6.2318-2320.2004 15155244 PMC415565

[9] Cunningham ME, Davidson-Wright J, Chilton M, Jones M, Pandey RK, Sheri A, et al Pan-genotypic anti-HCV activity of SB 9200 assessed in the ‘capture-fusion’ replication assay. Hepatology. 2013;58((S1)):92A–207A, Abstract 473.

[10] ClinicalTrials.gov. 2013. A multiple ascending dose phase I study of SB 9200 in treatment naïve adults with chronic hepatitis C infection. ClinicalTrials.gov identifier: NCT01803308.

[11] Iyer RP, Sheri A, Pandey RK, Padmanabhan S, Korba BE, Bose S, et al. Activation of intracellular viral sensors by the anti-hepatitis Agent SB 9200 –Implications for broad-spectrum antiviral activity. Abstracts of the 27th International Conference on Antiviral Research; 2014, May 12–16; Raleigh, NC; International Society for Antiviral Research, Washington, DC 2014. 2014.

[12] Korolowicz KE, Iyer RP, Czerwinski S, Suresh M, Yang J, Padmanabhan S, et al Antiviral Efficacy and Host Innate Immunity Associated with SB 9200 Treatment in the Woodchuck Model of Chronic Hepatitis B. PLoS One. 2016;11(8):e0161313 10.1371/journal.pone.0161313 27552102 PMC4995001

[13] Takeuchi O, Akira S. Pattern recognition receptors and inflammation. Cell. 2010;140(6):805–20. 10.1016/j.cell.2010.01.022 20303872

[14] Sato S, Li K, Kameyama T, Hayashi T, Ishida Y, Murakami S, et al The RNA Sensor RIG-I Dually Functions as an Innate Sensor and Direct Antiviral Factor for Hepatitis B Virus. Immunity. 2015;42(1):123–32. 10.1016/j.immuni.2014.12.016 25557055

[15] Menne S, Cote PJ. The woodchuck as an animal model for pathogenesis and therapy of chronic hepatitis B virus infection. World J Gastroenterol. 2007;13(1):104–24. 17206759 10.3748/wjg.v13.i1.104 PMC4065868

[16] Coughlin JE, Pandey RK, Padmanabhan S, O'Loughlin KG, Marquis J, Green CE, et al Metabolism, pharmacokinetics, tissue distribution, and stability studies of the prodrug analog of an anti-hepatitis B virus dinucleoside phosphorothioate. Drug Metab Dispos. 2012;40(5):970–81. 10.1124/dmd.111.044446 22328581 PMC3336794

[17] Hornbuckle WE, Graham ES, Roth L, Baldwin BH, Wickenden C, Tennant BC. Laboratory assessment of hepatic injury in the woodchuck (Marmota monax). Lab Anim Sci. 1985;35(4):376–81. 2864472

[18] Berraondo P, Di Scala M, Korolowicz K, Thampi LM, Otano I, Suarez L, et al Liver-directed gene therapy of chronic hepadnavirus infection using interferon alpha tethered to apolipoprotein A-I. J Hepatol. 2015;63(2):329–36. 10.1016/j.jhep.2015.02.048 25772035 PMC4508219

[19] Menne S, Butler SD, George AL, Tochkov IA, Zhu Y, Xiong S, et al Antiviral effects of lamivudine, emtricitabine, adefovir dipivoxil, and tenofovir disoproxil fumarate administered orally alone and in combination to woodchucks with chronic woodchuck hepatitis virus infection. Antimicrob Agents Chemother. 2008;52(10):3617–32. 10.1128/AAC.00654-08 18676881 PMC2565907

[20] Cote PJ, Roneker C, Cass K, Schodel F, Peterson D, Tennant B, et al New enzyme immunoassays for the serologic detection of woodchuck hepatitis virus infection. Viral Immunol. 1993;6(2):161–9. 10.1089/vim.1993.6.161 8216715

[21] Yoneyama M, Kikuchi M, Natsukawa T, Shinobu N, Imaizumi T, Miyagishi M, et al The RNA helicase RIG-I has an essential function in double-stranded RNA-induced innate antiviral responses. Nat Immunol. 2004;5(7):730–7. 10.1038/ni1087 15208624

[22] Sabbah A, Chang TH, Harnack R, Frohlich V, Tominaga K, Dube PH, et al Activation of innate immune antiviral responses by Nod2. Nat Immunol. 2009;10(10):1073–80. 10.1038/ni.1782 19701189 PMC2752345

[23] Takeuchi O, Akira S. Innate immunity to virus infection. Immunol Rev. 2009;227(1):75–86. 10.1111/j.1600-065X.2008.00737.x 19120477 PMC5489343

[24] Korba BE, Cote PJ, Menne S, Toshkov I, Baldwin BH, Wells FV, et al Clevudine therapy with vaccine inhibits progression of chronic hepatitis and delays onset of hepatocellular carcinoma in chronic woodchuck hepatitis virus infection. Antivir Ther. 2004;9(6):937–52. 15651753

[25] Liu J, Zhang E, Ma Z, Wu W, Kosinska A, Zhang X, et al Enhancing virus-specific immunity in vivo by combining therapeutic vaccination and PD-L1 blockade in chronic hepadnaviral infection. PLoS Pathog. 2014;10(1):e1003856 10.1371/journal.ppat.1003856 24391505 PMC3879364

[26] Menne S, Tumas DB, Liu KH, Thampi L, AlDeghaither D, Baldwin BH, et al Sustained Efficacy and Seroconversion with the Toll-Like Receptor 7 Agonist GS-9620 in the Woodchuck Model of Chronic Hepatitis B. J Hepatol. 2015. 10.1016/j.jhep.2014.12.026 PMC4439359 25559326

[27] Fletcher SP, Chin DJ, Gruenbaum L, Bitter H, Rasmussen E, Ravindran P, et al Intrahepatic transcriptional signature associated with response to interferon-α treatment in the woodchuck model of chronic hepatitis B. PLOS Pathogens 2015;(in press). 10.1371/journal.ppat.1005103 PMC4564242 26352406

[28] Fletcher SP, Chin DJ, Ji Y, Iniguez AL, Taillon B, Swinney DC, et al Transcriptomic analysis of the woodchuck model of chronic hepatitis B. Hepatology. 2012;56(3):820–30. 10.1002/hep.25730 22431061 PMC3401284

[29] Wieland S, Thimme R, Purcell RH, Chisari FV. Genomic analysis of the host response to hepatitis B virus infection. Proc Natl Acad Sci U S A. 2004;101(17):6669–74. 10.1073/pnas.0401771101 15100412 PMC404103

[30] Nakagawa S, Hirata Y, Kameyama T, Tokunaga Y, Nishito Y, Hirabayashi K, et al Targeted induction of interferon-lambda in humanized chimeric mouse liver abrogates hepatotropic virus infection. PLoS One. 2013;8(3):e59611 10.1371/journal.pone.0059611 23555725 PMC3610702

[31] Fletcher SP, Chin DJ, Cheng DT, Ravindran P, Bitter H, Gruenbaum L, et al Identification of an intrahepatic transcriptional signature associated with self-limiting infection in the woodchuck model of hepatitis B. Hepatology. 2013;57(1):13–22. 10.1002/hep.25954 22806943 PMC3525799

[32] Colonno RJ, Genovesi EV, Medina I, Lamb L, Durham SK, Huang ML, et al Long-term entecavir treatment results in sustained antiviral efficacy and prolonged life span in the woodchuck model of chronic hepatitis infection. J Infect Dis. 2001;184(10):1236–45. 10.1086/324003 11679911

[33] Genovesi EV, Lamb L, Medina I, Taylor D, Seifer M, Innaimo S, et al Efficacy of the carbocyclic 2'-deoxyguanosine nucleoside BMS-200475 in the woodchuck model of hepatitis B virus infection. Antimicrob Agents Chemother. 1998;42(12):3209–17. 9835516 10.1128/aac.42.12.3209 PMC106024

[34] Sarasin-Filipowicz M, Oakeley EJ, Duong FH, Christen V, Terracciano L, Filipowicz W, et al Interferon signaling and treatment outcome in chronic hepatitis C. Proc Natl Acad Sci U S A. 2008;105(19):7034–9. 10.1073/pnas.0707882105 18467494 PMC2383932

[35] Chen L, Borozan I, Sun J, Guindi M, Fischer S, Feld J, et al Cell-type specific gene expression signature in liver underlies response to interferon therapy in chronic hepatitis C infection. Gastroenterology. 2010;138(3):1123–33 e1–3. 10.1053/j.gastro.2009.10.046 19900446

[36] Gao B, Hong F, Radaeva S. Host factors and failure of interferon-alpha treatment in hepatitis C virus. Hepatology. 2004;39(4):880–90. 10.1002/hep.20139 15057887

[37] Dunn C, Peppa D, Khanna P, Nebbia G, Jones M, Brendish N, et al Temporal analysis of early immune responses in patients with acute hepatitis B virus infection. Gastroenterology. 2009;137(4):1289–300. 10.1053/j.gastro.2009.06.054 19591831

